# TDP‐43 related mis‐splicing and decreased protein levels of synapse related genes are associated with cognitive impairment in chronic traumatic encephalopathy

**DOI:** 10.1002/alz70855_096742

**Published:** 2025-12-23

**Authors:** Thor D. Stein, Nurgul Aytan, Jenny A Empawi, Raymond Nicks, Jonathan D Cherry, Victor E. Alvarez, Michael L. Alosco, Jesse Mez, Adam Thomas Labadorf, Ann C. McKee, Xiaoling Zhang, Daniel A Mordes

**Affiliations:** ^1^ Department of Pathology and Laboratory Medicine, Boston University Chobanian & Avedisian School of Medicine, Boston, MA, USA; ^2^ VA Bedford Healthcare System, Bedford, MA, USA; ^3^ Boston University Chronic Traumatic Encephalopathy Center, Boston University Chobanian & Avedisian School of Medicine, Boston, MA, USA; ^4^ Boston University, Boston, MA, USA; ^5^ Boston University Chobanian & Avedisian School of Medicine, Boston, MA, USA; ^6^ Boston University Chronic Traumatic Encephalopathy Chobanian and Avedisian School of Medicine, Boston, MA, USA; ^7^ Department of Pathology and Laboratory Medicine, Boston University School of Medicine, Boston, MA, USA; ^8^ Department of Neurology, Boston University School of Medicine, Boston, MA, USA; ^9^ Boston University Alzheimer's Disease Research Center, Boston, MA, USA; ^10^ VA Boston Healthcare System, Boston, MA, USA; ^11^ Boston VA Medical Center, Boston, MA, USA; ^12^ Department of Veterans Affairs Medical Center, Bedford, MA, USA; ^13^ Boston University Alzheimer's Disease Research Center, Boston University Chobanian & Avedisian School of Medicine, Boston, MA, USA; ^14^ Department of Neurology, Boston University Chobanian & Avedisian School of Medicine, Boston, MA, USA; ^15^ Biomedical Genetics, Department of Medicine, Boston University Medical School, Boston, MA, USA; ^16^ Department of Biostatistics, Boston University School of Public Health, Boston, MA, USA; ^17^ Bioinformatics Program, Boston University, Boston, MA, USA; ^18^ University of California San Francisco, San Francisco, CA, USA

## Abstract

**Background:**

Repetitive head impacts that occur in contact/collision sports, military service, and physical violence are associated with TDP‐43 pathology, and TDP‐43 inclusions are frequently present in the hippocampus and frontal cortex in chronic traumatic encephalopathy (CTE). Individuals with CTE and TDP‐43 inclusions are more likely to show severe cognitive impairment; however, the underlying mechanism is unknown. In amyotrophic lateral sclerosis (ALS) and frontotemporal lobar degeneration with TDP‐43 inclusions (FTLD‐TDP), TDP‐43 inclusions are associated with widespread gene mis‐splicing and cryptic exon expression. We hypothesized that TDP‐43 pathology in CTE similarly contributes to gene mis‐splicing, leading to selective protein loss and neurodegeneration.

**Methods:**

A total of 212 brain donors with a history of repetitive head impacts were examined for CTE and the presence and distribution of TDP‐43 inclusions. Clinical outcomes, including measures of cognitive impairment, were obtained from informants and medical records. Bulk RNA sequencing and SomaScan 7K aptamer‐based proteomics were performed on the dorsolateral prefrontal cortex. After excluding those with AD, FTLD‐TDP, or ALS, variable splicing events were determined using LeafCutter in those with CTE and TDP‐43 inclusions in the hippocampus or frontal cortex (CTE‐TDP) compared to CTE without TDP‐43 in those regions (CTE).

**Results:**

Out of 142 with CTE, 72 (51%) had TDP‐43 inclusions within the hippocampus, frontal cortex, or both (CTE‐TDP). Differential analysis revealed altered splicing within 836 genes in CTE‐TDP compared to CTE with a false discovery rate <0.01. Of these, 23 overlapped with previously identified mis‐spliced genes in ALS and FTLD‐TDP. Seven of these genes had protein levels measured with SomaScan proteomics, including CAMK2B, DLGAP4, EPB41L1, NCAM1, PTPRD, RAP1GAP, and SH3KBP1. Notably, protein levels of erythrocyte membrane protein band 4.1 like 1 (EPB41L1) and RAP1 GTPase‐activating protein (RAP1GAP) were significantly reduced in CTE‐TDP frontal cortex compared to CTE (*p*'s <0.01) and negatively correlated with both amygdala and hippocampal TDP‐43 inclusions. Furthermore, decreased levels of EPB41L1 and RAP1GAP were associated with dementia adjusting for age (*p*'s <0.05).

**Conclusions:**

Both EPB41L1 and RAP1GAP are involved in synaptic plasticity and signaling. Mis‐splicing and decreased protein expression of these synapse‐related genes may partially underlie the neurodegeneration and cognitive impairments in CTE with TDP‐43 inclusions.

